# Safety Considerations for Thermoplastic-Type Appliances Used as Orthodontic Aligners or Retainers. A Systematic Review and Meta-Analysis of Clinical and In-Vitro Research

**DOI:** 10.3390/ma13081843

**Published:** 2020-04-14

**Authors:** Anna Iliadi, Despina Koletsi, Spyridon N. Papageorgiou, Theodore Eliades

**Affiliations:** 1 Department of Biomaterials, School of Dentistry, National and Kapodistrian University of Athens, 11527 Athens, Greece; annaeliades@gmail.com; 2 Clinic of Orthodontics and Pediatric Dentistry, Center of Dental Medicine, University of Zurich, 8032 Zurich, Switzerland; d.koletsi@gmail.com (D.K.); snpapage@gmail.com (S.N.P.)

**Keywords:** orthodontic aligner, orthodontic retainer, clear aligner, thermoplastic, vacuum-formed, bisphenol-A, BPA, cytotoxicity, estrogenicity, systematic review

## Abstract

Use of thermoplastic material in orthodontics, either as aligner or as retainer appliances, is common practice and is likely to increase in the years to come. However, no systematic assessment on safety considerations of these adjuncts has been implemented up to date. The aim of this systematic review was to collectively appraise the existing evidence from both clinical and laboratory studies, on whether these appliances are associated with any estrogenic/cytotoxic effects or bisphenol-A (BPA) and monomer leaching. Eight electronic databases were searched with no limits in December 22, 2019, for published and unpublished research. Eligibility criteria comprised of studies of any design, describing use of any type of thermoplastic aligner. Study selection, data extraction and risk of bias (RoB) assessment was done independently, either in duplicate or confirmed by a second reviewer. Random effects meta-analyses of weighted mean differences (WMD) with associated 95% Confidence Intervals (CIs) were planned. Quality of the evidence was evaluated with Grading of Recommendations Assessment, Development and Evaluation (GRADE). A total of 58 articles were initially identified, while 5 were included in qualitative synthesis and 2 of those contributed to the quantitative syntheses. Four studies were in-vitro, while one was a randomized controlled trial; all assessed some type of orthodontic aligner or retainer, either as-received or retrieved. Risk of bias recordings ranged between unclear and high for all studies. Proliferation induction capacity of thermoplastic appliances’ eluents on MCF-7 cells failed to be confirmed compared to beta-estradiol (2 studies: 5% *v*/*v*, WMD: −182.08; 95% CI: −198.83, −165.33; *p*-value < 0.001; and 20% *v*/*v*, WMD: −184.53; 95% CI: −206.17, −162.88; *p*-value < 0.001). No cytotoxic activity was detected as well. In addition, although evidence from in-vitro studies was indicative of no traceable detection of BPA or other monomers, the findings from a single clinical trial were allied to increased levels of BPA in whole stimulated saliva, after up to 30 days of thermoplastic retainer usage, compared to standard Hawley retainer. The quality of the evidence overall was low to medium. Current data from in-vitro research are indicative of an absence of an estrogenic or cytotoxic effect of thermoplastic aligners or retainers. Regarding BPA or monomer release, evidence from clinical and laboratory studies appear inconsistent.

## 1. Introduction

Aligner orthodontic therapy has been in the spotlight of the recent years, partially due to potentially aggressive promotion policies of companies, manufacturers and stakeholders but also due to patients’ perception for ‘invisible’ orthodontics and aesthetic considerations. It is noteworthy that the increase in the use of aligners as the sole means or adjunct for tooth movement and full orthodontic treatment has reached considerable amounts [1]. The reported increase in the use of aligners by orthodontists in the United States within a period of 6 to 7 years has reached 15%, while the number of patients receiving treatment with aligners has been nearly duplicated [2].

As a rather “new” adjunct for the orthodontist but with upcoming massive use, aligner treatment should be subject to assessment in terms of its efficacy and safety for clinical operation. The latest meta-analysis report on the efficacy of aligners compared to the gold standard of fixed appliances, allowing for individual study caveats, revealed that aligner treatment cannot be considered equally effective to braces at present, while their use is associated with worse treatment outcome [3,4]. However, with regard to safety considerations on the use of aligners, no similar comprehensive effort has been attempted. Sporadic reports, mainly in vitro, have examined potential cytotoxic effects of these appliances [5,6]. Recently, an in-vivo study on retainers has documented increased levels of bisphenol-A (BPA) release in saliva of patients using vacuum-formed thermoplastic adjuncts during the retention phase, compared to the use of classic Hawley-type appliances [7]. Thermoplastic orthodontic adjuncts as retainers cannot be considered inferior to the classic type-Hawley retainers at present and in view of the available evidence on certain perspectives, such as stability or attainment of proper oral hygiene [8]. Moreover, they have also been associated with increased patients’ compliance [9].

Bisphenol-A constitutes a well-established monomer used during the production of a wide variety of orthodontic materials, for manufacturing of a range of polymers, such as resin composites, esthetic brackets, elastomeric ligatures but also thermoplastic aligner or retainer systems. It is a raw chemical used as a precursor of bisphenol A-glycidyl methacrylate (Bis-GMA) during its manufacturing phase [10]. Concerns regarding BPA release from clinically usable materials have been oriented towards identifying on one part whether these orthodontic materials bear the potential to exhibit estrogenic or other cytotoxic actions; on the other hand, towards detecting bisphenol-A release or residual monomer leaching [5,10,11,12].

Therefore, the aim of the present systematic review was to comprehensively collect and appraise the existing evidence from either in-vitro or clinical research, if the latest exists, as to whether thermoplastic aligner or retainer use in orthodontics has been associated with increased levels of BPA release or residual monomer leaching and also to record any profound cytotoxic or estrogenic effects. The null hypothesis was that aligners/retainers cannot be linked to any of the above-mentioned activities.

## 2. Materials and Methods

### 2.1. Protocol Registration and Reporting

The protocol of this study has been registered in the Open Science Framework as of January 20, 2020 (https://osf.io/9wsy2/) [13]. Reporting has been conducted in line with the Preferred Reporting Items for Systematic Reviews and Meta-Analyses (PRISMA) guidelines [14,15].

### 2.2. Eligibility Criteria

Eligibility criteria for study selection have been schemed as follows:

1. Study design: any type of study, irrespective of the design, that is, randomized controlled trial, prospective clinical trial, retrospective cohort, in-vitro, pre-clinical studies, irrespective of the groups under comparison.
2. Participants: patients undergoing orthodontic treatment with aligners or wearing retainers after the fulfillment of orthodontic treatment are considered eligible for clinical studies. For in-vitro/pre-clinical research, any type of thermoplastic aligner either retrieved or as-received was included.
3. Intervention: any type of thermoplastic aligner/retainer (retrieved/as-received) used in clinical or in-vitro research. These include all types or material thickness, type, activation with/without attachments.
4. Comparator: any type of thermoplastic aligner/retainer used as comparator group or even studies without a comparator/control group involved.
5. Outcome: BPA-release, any type of monomer release including BisGMA, triethylene glycol dimethacrylate (TEGDMA) and byproducts. Also, outcomes indicating cell proliferation activity, including but not confined to MCF-7 breast cancer cells or other estrogen-responsive cells.
6. Exclusion criteria: case studies, studies involving raw thermoplastic materials/sheets used for aligners/retainers but not pressed and formed as aligners/retainers for clinical use.

### 2.3. Search Strategy

Electronic searching was conducted within 8 databases including published and unpublished research, with no language/date restriction or other filter modifications (Appendix A Table A1). The date of search within all databases was December 22, 2019. The respective databases were—Medline via PubMed, Scopus, Cochrane Central Register of Controlled Trials (CENTRAL), Cochrane Database of Systematic Reviews (CDSR) and Google Scholar. Moreover, unpublished literature was searched in the Open Grey, the ClinicalTrials.gov (www.clinicaltrials.gov), the National Research Register (www.controlled-trials.com). Hand searching of the eligible for inclusion articles was employed for any additional potential inclusion and authors of the included papers were contacted when in need to clarify on data extraction or data curation. Keywords involved “thermoplastic aligner,” “vacuum-formed retainer,” “bisphenol-A release,” “BPA release,” “monomer release,” “estrogenicity.”

Titles and abstracts of papers were extracted first, followed by full text assessment. Eligibility assessment was performed independently and in duplicate by two authors (A.I., D.K.), while any disagreements were settled through discussion and a consensus was reached with the involvement of a third reviewer (T.E.).

### 2.4. Data Extraction

Data extraction was employed in pre-piloted standardized forms by a single reviewer (A.I.), not blinded to study origin or author identity, while all entries were confirmed by a second investigator (D.K.). Specifically, information entries were related to study identity, study design, sample size, intervention, comparators, outcomes, method of analysis.

### 2.5. Risk of Bias Assessment within Individual Studies

Risk of bias assessment was performed independently and in duplicate by two authors (A.I., D.K.). Any disagreements were settled after consultation with a third author (T.E.). For the potentially included randomized controlled trials, the updated Cochrane RoB 2.0 tool was planned to be used [16] (Sterne 2019). A modification of this tool was utilized for the included in-vitro/pre-clinical studies, as no pre-determined guidelines to assess the risk of bias exist and in order to incorporate specific important elements that would help identify the presence of potential bias. These include selection bias [17,18], performance bias [19], attrition bias [20] and reporting issues [21,22].

### 2.6. Summary Measures and Data Synthesis

Prior to any decision to quantitatively pool together data from individual studies, clinical heterogeneity was examined in terms of individual study settings, trial or laboratory conditions, inclusion criteria or methods of analyses. If possible, statistical heterogeneity was planned to be examined, first visually, through inspection of the confidence bounds within the forest plots, as well as statistically, as indicated by a *p*-value below the level of 10% for the test (*p* < 0.10) [23]. I^2^ test for homogeneity was also planned to be undertaken.

Random-effects meta-analyses were planned as they were considered more appropriate to incorporate individual study findings if possible. In view of the anticipated continuous nature of the expected outcomes, treatment effects were planned to be calculated through pooled weighted mean differences (WMD) or mean differences (MD) for single studies with associated 95% Confidence Intervals (95% CIs).

### 2.7. Risk of Bias across Studies

If more than 10 studies were included in meta-analyses, publication bias was to be explored through standard funnel plots [24] and Egger’s regression test [25].

### 2.8. Assessment of the Quality of the Evidence

The Grading of Recommendations Assessment, Development and Evaluation (GRADE) were implemented to assess the overall quality of evidence as formulated by the interventions and the outcomes under evaluation [26,27]. According to GRADE the overall body of evidence is rated as high, moderate, low and very low. The ratings, with regard to the likelihood for a change in our confidence in the estimated effect, range from very unlikely to very likely. In addition, when the overall quality of the body of evidence is very low, then any estimated effect is particularly uncertain. Assessment of the body of evidence and the initial starting-up level depends on the study design. In terms of randomized designs, which present a theoretically ‘high’ quality of the evidence, assessment is made following the domains: risk of bias, inconsistency, indirectness, imprecision and publication bias. For the first 4 domains the quality of evidence may be downgraded on the basis of either ‘serious’ or ‘very serious’ risks (1 or 2 levels respectively); publication bias may either be suspected or undetected (2 levels). For non-randomized/observational designs, which theoretically start from a ‘low’ level of evidence, there are 3 perspectives for upgrade: a large effect, a plausible residual confounding that may alter the effect or a dose-response gradient. The level of evidence may be upgraded by 1 or 2 levels (large effect) or 1 level (plausible confounding, does-response gradient).

All analyses were undertaken in Stata version 15.1 software (StataCorp, College Station, TX, USA).

## 3. Results

### 3.1. Search Details

**Figure 1:**
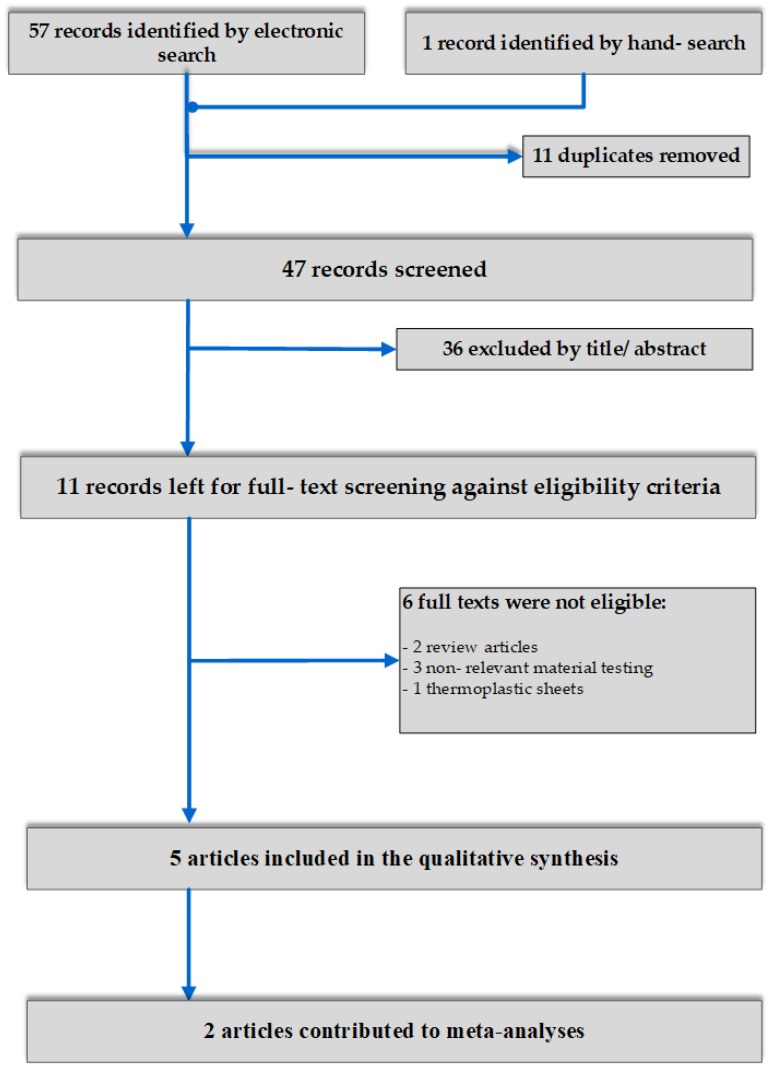
Flow diagram of study selection.

A total number of 58 results were initially retrieved after application of electronic search strategies. After duplicate exclusion and screening first by title and subsequently by abstract, eleven articles were left for full-text assessment, leaving 5 to be included in the qualitative synthesis [5,6,7,28,29]. Of these, 2 contributed to meta-analyses for different outcomes [5,6] (Figure 1).

### 3.2. Study Design and Characteristics

Detailed information about study design and characteristics is presented in Table 1.

Studies presented a variable origin background, with authorship affiliation to Europe, Asia, the United States as well as the United Arab Emirates. The first study in the field dates back to 2004 [29], while in the last five years only two research papers described the results of safety considerations regarding thermoplastic appliances used for orthodontic purposes either in the course of treatment or in the retention phase [6,7].

Only one study presented a clinical trial design (randomized controlled trial, RCT) [7], while the remaining 4 examined outcomes on in-vitro tested materials in the laboratory [5,6,28,29]. Three studies tested on both as-received (or-pretreatment) and retrieved (after use) material [6,7,29], while 2 only on newly received aligners [5,28]. Sample sizes for aligner units ranged from 6 to 45, while when aligners’ eluents were examined for cell response or monomer/BPA leaching, the respective contributing units were effectively larger, constituting up to 96 eluent samples from respective micro-wells examined.

Two studies searched for the potentially estrogenic effect of aligners’ eluents linked to their capacity to induce proliferation of breast cancer cells (MCF-7), using either beta-estradiol [5,6] or BPA [5] as a positive control for comparison. The latter report [5] also searched for the potential of eluents of aligners to produce proliferative activity of human gingival fibroblasts, thus indicating a cytotoxic dynamic. All immersion media concentration used, had a range between 5% vol/vol and 20% vol/vol. The study of Schuster 2004 [29] focused on broader outcomes of structural conformation of the material as well as on in-vitro substance leaching. BPA leaching was also the reported outcome for the study of Kotyk 2014 [28], which reported on a wide range of dental materials including thermoplastic retainers. The primary outcome of the sole clinical trial included in this review was whole stimulated saliva BPA levels after the use of three-types of retainers including a thermoplastic vacuum formed one, after a consecutive time-points of 1 day, 7 days and 30 days of aligner use [7].

Technical analysis methodology of the included studies pertained to a spectrum of tools, in close proximity with the reported outcome. These included, estrogenicity and/or cytotoxicity assays for cell culture [5,6], gas chromatography–mass spectrometry (GS-MS) [28,29] and high-performance liquid chromatography (HPLC) [7].

### 3.3. Risk of Bias within Studies

**Figure 2:**
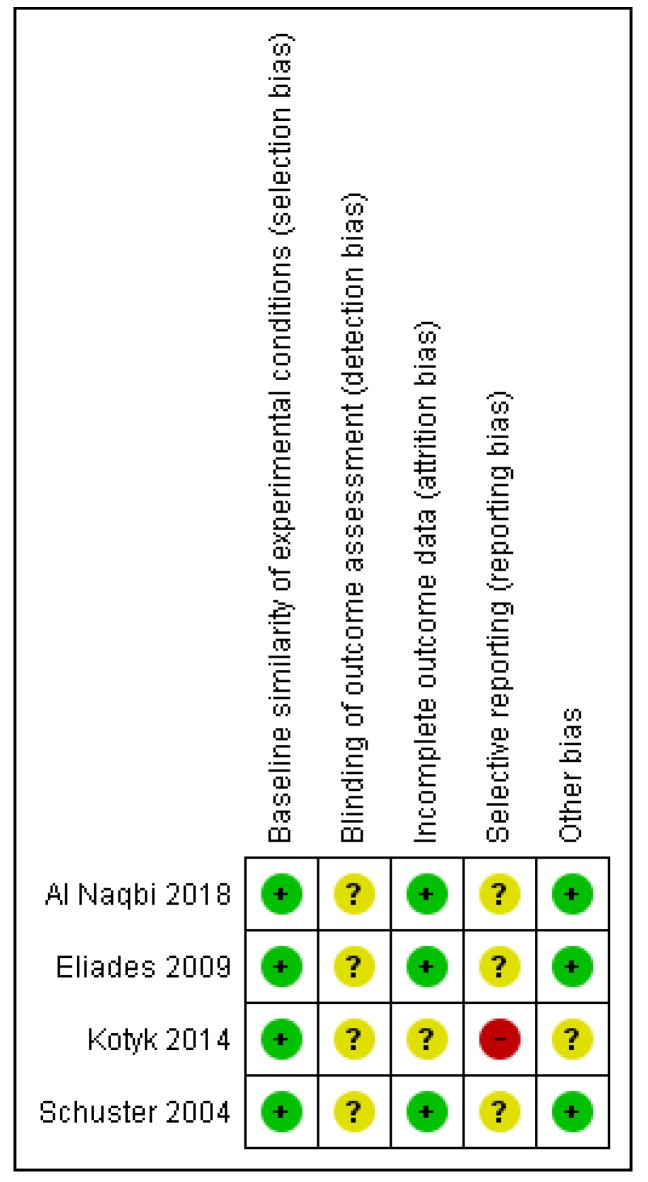
Risk of bias summary: review authors’ judgements about each risk of bias item for each included study. The green plus sign indicates low risk of bias; the yellow question-mark, unclear and the red minus, high.

Internal validity of the included studies ranged from unclear to high risk of bias for the in-vitro designs [5,6,28,29] (Figure 2), while the sole RCT was rated as high risk of bias overall [7] (Table 2; Table S1).

Breakdown to specific risk of bias tool domains for the in-vitro studies, revealed as the most afflicted domain, the potential lack of blinding of the outcome assessors, directly linked to detection bias, with no information provided in any of the four in-vitro design studies. In addition, no data implied the existence of a pre-registered protocol of the study, thus raising concerns about selective reporting overall. Contrary, baseline similarity of the experimental groups was considered adequate for all studies, while retaining of the materials throughout the experiment was also reasonable. For the RCT [7], the weakest domains pertained to randomization issues not adequately reported and possibly conducted, as well as concerns related to participants’ or investigators’ awareness of the intervention that might have potentially led to non-compliance or deviations from the intended intervention. Some concerns were also raised for the possibility of this study being prone to detection bias or selective reporting.

### 3.4. Effects of Interventions, Meta-Analyses and Additional Analyses

Overall, two articles contributed to meta-analyses, while there was a range of outcomes recorded across the included studies. Quantitative data from mathematical syntheses, as well as from individual single study findings are shown in Table 3.

**Figure 3:**
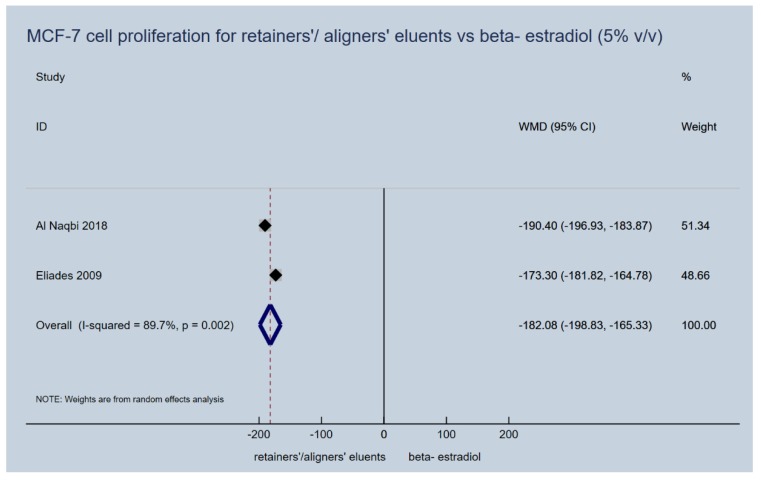
Random effects meta-analysis on the effect of thermoplastic appliances’ eluents versus beta-estradiol (5% *v*/*v*) to induce proliferation activity of MCF-7 cells.

**Figure 4:**
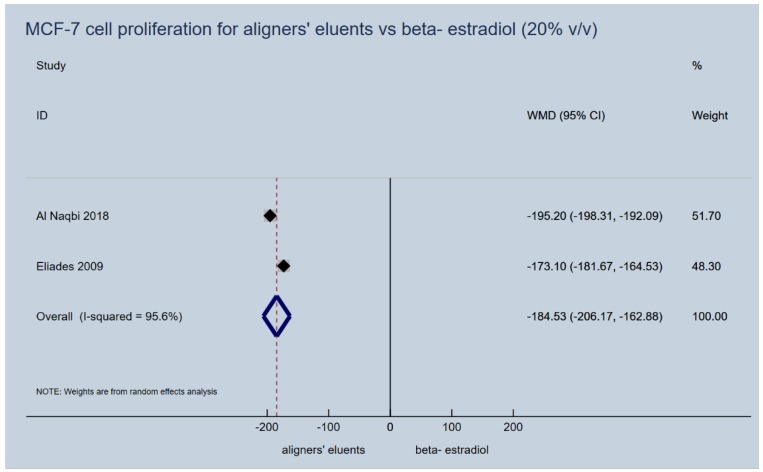
Random effects meta-analysis on the effect of thermoplastic appliances’ eluents versus beta-estradiol (20% *v*/*v*) to induce proliferation activity of MCF-7 cells.

Proliferation induction capacity of aligners’ (retainers’) eluents on MCF-7 cells failed to be confirmed for any of the upper or lower extreme immersion media concentrations (5% vol/vol and 20% vol/vol), that were used in the laboratory studies. They presented a significantly lower cancer cell proliferation dynamic, as expressed by the percentage of the control vehicle used in vitro, compared to beta-estradiol (5% *v*/*v*, WMD: −182.08; 95% CI: −198.83, −165.33; *p*-value < 0.001; Figure 3 and 20% *v*/*v*, WMD: −184.53; 95% CI: −206.17, −162.88; *p*-value < 0.001; Figure 4). In essence, the proliferative activity of the appliances’ eluents were in the same levels or even lower of those recorded for the control normal saline vehicle, while this was not the case for beta-estradiol (Table 3).

Similarly, the results of a single study [5] that used BPA as an active control compared to aligners’ eluents MCF-7 cell proliferation dynamics, were in the same line as with beta-estradiol described above (5% *v*/*v*, MD: −74.1; 95% CI: −78.8, −69.4; *p*-value < 0.001 and 20% *v*/*v*, MD: −73.9; 95% CI: −78.7, −69.1; *p*-value < 0.001) (Table 3). Cytotoxic activity based on the potential for proliferation of human gingival fibroblasts, as reported in the same single study, was also not detected for aligners’ eluents and appeared even lower that the control normal saline. The single studies of Kotyk 2014 [28] and Schuster 2004 [29], did not report any measurable traces of either BPA or residual monomers and oxidative byproducts for any thermoplastic aligner/retainer under study.

With regard to the single RCT included [7], there was evidence that vacuum-formed retainers were associated with increased levels of BPA in whole stimulated saliva, within a period of 30 days evaluation (Table 3). Specifically, after 7 days of retainer wear, BPA levels (in ppm) in saliva were significantly higher for vacuum-formed retainers compared to both heat-cured common type Hawley appliances (MD: 2.38; 95% CI: 1.47, 3.29; *p*-value < 0.001), as well as chemically-cured Hawley retainers (MD: 2.38; 95% CI: 1.47, 3.29; *p*-value < 0.001). Following, there was a downstream reduction of the effect after 30 days of patients’ use but still remained significantly higher in those allocated to vacuum-formed versus heat-cured (MD: 0.20; 95% CI: 0.16, 0.25; *p*-value < 0.001), as well as chemically-cured Hawley retainers (MD: 0.20; 95% CI: 0.15, 0.24; *p*-value < 0.001).

### 3.5. Risk of Bias across Studies

Publication bias and small-study effects could not be explored, in view of the limited number of studies included.

### 3.6. Quality of the Evidence

The assessment of the quality of the evidence for estrogenicity related issues and induction of cancer cell proliferation in-vitro, revealed that the quality of the evidence was moderate. Justification of specific grading is presented in Table 4. Most importantly the level of evidence was downgraded due to heterogeneity related factors, while it was upgraded due to an observed large pooled estimated effect. In addition, the quality of the evidence was rated as low for BPA and cancer cell proliferation in-vitro. Further downgrading was implemented due to the assumed imprecision of the estimated effect, as only one study actually contributed (Table 4).

Regarding the BPA levels of different types of retainers after clinical use for up to 30 days, the quality of the evidence was graded as low, mainly due to serious shortcomings related to methodological limitations or risk of bias in certain domains of the included RCT (Table S2).

## 4. Discussion

### 4.1. Summary of the Evidence

In view of the increasing use of thermoplastic aligners either as orthodontic treatment adjuncts for “invisible” orthodontics or as passive appliances used at the retention phase, this systematic review comes timely for a comprehensive assessment on safety considerations after quite a few years of clinical use. As evidence from in-vitro studies seems to accrue, albeit within a limited speed rate, data from clinical trials on safety of thermoplastic appliances used for orthodontic-related reasons, currently lags behind; Notwithstanding this, physical and mechanical properties of thermoplastic adjuncts prior to or after intraoral service, have gained a considerably higher amount of interest in the scientific community, allied with their clinical use, with material conformation and thermoforming effect being the most eminent prognostic factors for the appliances’ properties’ variability [30,31]. On safety grounds, with regard to BPA leaching, only one high risk of bias and low quality of the evidence trial has been published up to date.

Concerns on BPA release from everyday consumption products and the effects on human organs dates back to 1980s or 1990s, when researchers documented suspicious for estrogenic activity doses close but even lower than the nationally set of baseline dose of 50μg per weight, by the United States (US) Environmental Protection Agency (EPA) [32]. In essence, animal and in-vitro studies had underpinned the effect of the monomer on tissues and cells, on par with 17-beta estradiol, a natural estrogen. As such, bisphenol-A has been reported to act through mimicking the natural estrogen and to allow for a number of estrogenic or cytotoxic effects to take place within live organisms. Some of these effects include insulin tolerance through the disruption of beta-pancreatic cells’ physiologic activity [33], disruption of physiologic prostate development or increased risk for prostate cancer in men and breast cancer in women [34,35] or alterations in the physiology of mammary gland development [36].

Although a number of studies have raised early concerns about BPA release or monomer leaching related not only to dental adhesives/resins [37,38] but also to orthodontic materials in use [29,39,40], evidence on safety considerations of thermoplastic aligners or retainer adjuncts is not that straightforward and abundant.

Findings from the laboratory studies seem to agree that there is no discernable cancer cell proliferation inducing capacity of thermoplastic orthodontic adjuncts, either of as-received (new) [5,6] or of patient retrieved [6]. Effectively this was the case, when tested against the standard estradiol dynamics for cell proliferation or even for the BPA effects on such cells. However, when retrieved retainers were examined, they had been previously undergone some kind of sterilization procedure which might have masked a potentially altered effect on the dynamics for cell proliferation. Nevertheless, such an effect of a sterilization procedure was not evident for as-received appliances. On the other side, findings from the sole RCT on the topic [7], revealed a clearly documented effect of vacuum-formed retainers over standard Hawley appliances, on the promotion of BPA leaching, primarily in short term after usage but also within the limit of 1 month, that was actually the longest follow-up period examined. Interestingly, such an association had not been detected by two earlier in-vitro studies on the topic [28,29], which did not report anything on detection of BPA or leaching of other residual monomers or oxidative byproducts, at least at a considerable amount.

### 4.2. Findings in Context

A critical step towards interpretation of the existing level of evidence and its placement in the appropriate context would be to acknowledge the potentially different picture of a thermoplastic appliance’s behavior within the oral cavity and under strict experimental conditions [10]. Clinical use of orthodontic aligners or retainers is subject to unavoidable and unpredictable mastication stress, variations in oral cavity pH and/or temperature, which, allied with the patients’ cooperation in appliances’ wear, may form a variable entirety that may not be captured by laboratory conditions and instrumental assessment. A recent study on the surface roughness and mechanical properties of commercially available aligners after short term clinical use has revealed significant material properties’ differences compared to non-aged material, as well as appliance wear even after only one week of service [41]. It may be speculated that this is mostly important for aligner-type adjuncts that may actively impose certain amounts of stress levels to induce a desirable tooth movement. On the other hand, passive vacuum-formed thermoplastic retainers may not be subject to increased levels of intraoral stress and one might argue against the high potential of intraoral wear, thus afflicting with safety considerations on monomer leaching intraorally; notwithstanding, the duration of a retainer wear may probably be by far more elongated that that of an aligner, allowing a non-negligible amount of intraoral mastication forces and dentition-retainer interaction to take place, especially if one considers the occlusal contacts.

On top of that, the inclusion of resin-based attachments to aligner therapy in an attempt to guide tooth movement and orchestrate tooth displacement more effectively, has become almost universal among the users of such treatment alternatives [42,43]. Apparently, the inclusion of such auxiliaries to a full-case orthodontic treatment with aligners gives rise to certain emerging issues: first, the inclusion of additional adhesive/resin based materials, of variable number, size and scheme or shape, which present themselves as “extensions” to natural dentition bounds, are possibly likely to demonstrate estrogenic activity through BPA/monomer release in the oral cavity. This may be employed both through intraoral aging of the material but also through production of aerosol compounds at the removal stage at the end of the aligner treatment [44,45]. Aerosol creation and simultaneous inhalation has been regarded detrimental, if deposited to the respiratory system [46]. Second, attachment-aligner interface is likely to create constant abrasion of attachment materials following multiple rounds of placement and removal of the aligners, even within a single day of service. Such practices are most probably likely to create material particles released within the oral environment. In the light of the aforementioned considerations, any further inference on the potentially estrogenic or cytotoxic effects of contemporary treatment with aligners may only be speculative, at present. Further research in the field is more than crucial.

### 4.3. Strengths and Limitations

Although we allowed for a wide variety of types of study designs to satisfy eligibility criteria, the final number of included studies was limited in view of the paucity of related research; however this reflects the identity and level of existing evidence up to date. Methodological limitations of the included studies are certainly not to be neglected and any individual study interpretation or summary conclusion should be considered under the prism of the identified shortcomings.

Our study protocol was openly registered a priori, search strategy involved eight databases including unpublished theses/studies identified within registries, while the conduct and reporting of the review followed well-stablished guidelines [14,15] and included a formal assessment of the quality of the evidence (GRADE) [26,27]. It was also straightforward that the existing research studies were confined almost uniquely to in-vitro research projects that aimed to assess the BPA leaching and the potential for simulation of cancer cell proliferation, following estrogenic and cytotoxic actions.

## 5. Conclusions

Overall, no estrogenic or cytotoxic effect of the thermoplastic appliances could be confirmed based on limited preliminary evidence from in-vitro studies, while their effect on monomer or BPA release across both in-vitro and clinical evidence remains ambiguous. As the current picture of data and available evidence remains obscure and often inconsistent, the most promising approach would be to welcome new laboratory studies and most importantly new clinical trials, to the highest level of conduct and reporting. This would allow for solid and robust inferences for clinical decision making, which we are currently missing.

## Figures and Tables

**Table 1:** Data extraction information for included studies (origin, study design, sample size, technical analysis methodology, comparisons and outcomes).

Author	Origin/Design	Sample Size	Technical Analysis Method	Groups under Comparison	Outcomes
Al Naqbi, 2018 [6]	UAE, Switzerland, Greece (in-vitro)	n = 12 Vivera^®^ retainers, 6 for each of the two groups (48 aligner eluents per group)	Estrogenicity assays, two line cells (MCF-7, MDA-MB-231). Cells cultured in Dulbecco’s modified eagle medium (DMEM) supplemented with 10% fetal bovine serum, at 37 °C, in 5% carbon dioxide. Finally, the cells were detached using trypsin-citrate solution and counted in a Z1 Beckman-Coulter counter. (48-well, flat-bottomed microwells, with approximately 10,000 cells per well), received samples of aligner solution eluents	1. New (as-received) (n = 6) vs 2. Retrieved after 4w of use (12 h/day) (n = 6) (overall breakdown: Non sterilized (n = 2), Sterilized through gamma-irradiation (n = 5), Sterilized through autoclaving (n = 5)); b-Estradiol (β-E2) was used as positive control; Solutions, at concentrations: 5%, 10% and 20%	1. estrogenicity assessed by cell counting/proliferation (MCF-7, MDA-MB-231); note: no estrogenic action induced by either group of retainers
Eliades, 2009 [5]	Greece (in-vitro)	3 sets of aligners (Invisalign, Align tech^®^) n = 6 (3 maxillay, 3 mandibular) (96 aligner eluents per group)	Cytotoxicity: by a modification of the MTT (Sigma, St Louis, Mo) assay; Estrogenicity: assays involved 2 cell lines: MCF-7 and MDA-MB-231. (96-well, flat-bottomed microwells, with approximately 5000 cells per well), received samples of aligner solution eluents	Eluents were diluted to 5%, 10% and 20% vol/vol, normal saline solution served as negative control and b-estradiol (β-E2) and BPA was used as positive controls	1. cytotoxicity (optical density of human gingival fibroblasts); 2. estrogenicity assessed by proliferation of MCF-7 and MDA-MB-231; note: no cytotoxic or estrogenic effects detected
Kotyk, 2014 [28]	Canada (in-vitro)	8 retainer materials, cut into pieces of unspecified number	GC-MS	1. Prethermoformed Biocryl Essix 2. Thermoformed Biocryl Essix 3. Prethermoformed Biocryl Retainer 4. Thermoformed Biocryl Retainer 5. Prethermoformed Dentsply Raintree Essix 6. Thermoformed Dentsply Essix 7. Unused Invisalign aligner 8. Used Invisalign aligner	1. BPA concentration (ppm/gr); note: leached concentrations and masses of BPA only for Thermoformed Biocryl Retainer, only after 1 day immersion in artificial saliva
Raghavan, 2017 [7]	India (RCT)	n = 45	HPLC	1. Vacuum-formed retainer, n = 15; 2. Hawley (heat cure), n = 15; 3. Hawley retainer (chemical cure), n = 15; at 4 time points: T0 (before placement), T1 (1 hour), T2 (1 week), T3 (1 month)	1. BPA levels of simulated whole saliva; note: highest levels were detected in group 1, followed by group 3
Schuster, 2004 [29]	USA, Greece (in-vitro)	n = 10 samples of aligners (Invisalign, Align tech) before intraoral placement (as received) and after retrieval; n = 12 samples of same aligners after placement intraorally for 22 h, for 2 weeks	Reflection microscopy, FTIR, scanning electron microscopy, vickers hardness, GC-MS	1. before placement, n = 10; 2. after retrieval (2 weeks), n = 12	1. aligner morphological variation (reflection microscopy, FTIR, scanning electron microscopy, vickers hardness); 2. substance leaching (GC-MS); note: no residual monomers or oxidative byproducts detected

RCT, randomized controlled trial; β-Ε2, beta estradiol; BPA, bisphenol-A; MTT, 3-(4,5-dimethylthiazol-2-yl)-2,5-diphenyltetrazolium bromide; GS-MS, gas chromatography–mass spectrometry; HPLC, high-performance liquid chromatography; FTIR, Fourier transform infrared spectroscopy.

**Table 2:** Risk of bias of included randomized clinical trial with the RoB 2.0 tool.

Study	Randomization	Deviations from Intended Interventions	Missing Outcome Data	Measurement of the Outcome	Selection of the Reported Result	Overall
Raghavan 2017 [7]	High	High	Low	Some concerns	Some concerns	High

**Table 3:** Quantitative data from meta-analyses and individual single studies for related groups under comparison and outcomes. The minus sign (-) shows lower effect for the first reported group. Bold indicate statistically significant comparisons.

#	Study ID	Groups under Comparison Per Study (N, mean, SD)	Outcome	WMD or MD (95% CIs)	*p*-Value	Heterogeneity (I^2^%)
1	2 studies	(Al Naqbi 2018) Aligner eluents (48, 96.0, 3.5) vs E2 (48, 286.4, 22.8) (Eliades 2009) Aligner eluents (96, 85.6, 17.4) vs E2 (96, 258.9, 38.9)	MCF-7 proliferation (5% *v*/*v*), % percentage of control vehicle	−182.08 (−198.83, −165.33)	< 0.001	89.7
2	−	(Al Naqbi 2018) Aligner eluents (48, 101.5, 2.14) vs E2 (48, 296.7, 10.8) (Eliades 2009) Aligner eluents (96, 85.8, 17.9) vs E2 (96, 258.9, 38.9)	MCF-7 proliferation (20% *v*/*v*), % percentage of control vehicle	−184.53 (−206.17, −162.88)	< 0.001	95.6
3	1 study (Eliades 2009) [5]	Aligner Eluents (96, 85.6, 17.4) BPA (96, 159.7, 15.8)	MCF-7 proliferation (5% *v*/*v*), % percentage of control vehicle	−74.1 (−78.8, −69.4)	< 0.001	−
4	−	Aligner Eluents (96, 85.8, 17.9) BPA (96, 159.7, 15.8)	MCF-7 proliferation (20% *v*/*v*), % percentage of control vehicle	−73.9 (−78.7, −69.1)	< 0.001	−
5	1 study [Raghavan 2017] [7]	VFR (15, 2.38, 1.80) Hheat (15, 3.9 × 10^−4^, 0.89 × 10^−5^)	BPA levels in saliva (ppm)/7 days	2.38 (1.47, 3.29)	< 0.001	−
6	−	VFR (15, 2.38, 1.80) Hchem (15, 3.54 × 10^−3^, 0.50 × 10^−3^)	BPA levels in saliva (ppm)/7 days	2.38 (1.47, 3.29)	< 0.001	−
7	−	Hheat (15, 3.9 × 10^−4^, 0.89 × 10^−5^) Hchem (15, 3.5 × 10^−3^, 0.50 × 10^−3^)	BPA levels in saliva (ppm)/7 days	−0.0035 (−0.0037, −0.0032)	< 0.001	−
8	−	VFR (15, 0.20, 0.09) Hheat (15, 6.1 × 10^−4^, 14 × 10^−4^)	BPA levels in saliva (ppm)/30 days	0.20 (0.16, 0.25)	< 0.001	−
9	−	VFR (15, 0.20, 0.09) Hchem (15, 9.25 × 10^−3^, 2.4 × 10^−3^)	BPA levels in saliva (ppm)/30 days	0.20 (0.15, 0.24)	< 0.001	−
10	−	Hheat (15, 6.1 × 10^−4^, 14 × 10^−4^) Hchem (15, 9.25 × 10^−3^, 2.4 × 10^−3^)	BPA levels in saliva (ppm)/30 days	−0.009 (−0.010, −0.007)	< 0.001	−

N, number of patients/sample eluents; WMD, weighted mean difference; MD, mean difference; SD, standard deviation; E2, beta-estradiol; BPA, bisphenol-A; VFR, vacuum-formed retainer; Hheat, Hawley heat-cured; Hchem, Hawley chemically-cured.

**Table 4:** Summary of Findings Table and quality of the evidence regarding aligners’ eluents capacity to induce MCF-7 cell proliferation.

Aligners’ Eluents Compared to Beta-Estradiol/BPA for Estrogenicity and BPA Leaching (through Induction of MCF-7 Proliferation)				
Sample Population (Eluents): For Assessment of Estrogenicity and BPA Leaching Settings: In-Vitro Design Intervention: Aligners’ Eluents Comparison: Beta-Estradiol (E2)/or Bisphenol-A (BPA), as Positive Control				
Outcomes	Illustrative Comparative Risks * (95% CI)		No of Samples (Eluents) (Studies)	Quality of the Evidence (GRADE)
	Assumed Risk	Corresponding Risk	−	−
−	Beta-Estradiol/BPA	Aligners’ Eluents	−	−
MCF-7 cell proliferation (5% *v*/*v*), % of control vehicle/E2	−	The mean MCF-7 cell proliferation (5% *v*/*v*), as % of control vehicle in the intervention groups was 182.1 lower (198.8 lower to 165.3 higher)	288 (2 studies)	⊕⊕⊕⊝ moderate ^1,2,3^
MCF-7 cell proliferation (20% *v*/*v*), % of control vehicle/E2	−	The mean MCF-7 cell proliferation (20% *v*/*v*), as % of control vehicle in the intervention groups was 184.5 lower (206.2 to 162.9 lower)	288 (2 studies)	⊕⊕⊕⊝ moderate ^1,2,3^
MCF-7 proliferation (5% *v*/*v*), % of control vehicle/BPA	−	The mean MCF-7 proliferation (5% *v*/*v*), as % of control vehicle in the intervention groups was 74.1 lower (78.8 to 69.4 lower)	192 (1 study)	⊕⊕⊝⊝ low ^4,5,6^
MCF-7 cell proliferation (20% *v*/*v*), % of control vehicle/BPA	−	The mean MCF-7 cell proliferation (20% *v*/*v*), as % of control vehicle in the intervention groups was 73.9 lower (78.7 to 69.1 lower)	192 (1 study)	⊕⊕⊝⊝ low ^4,5,6^
GRADE Working Group grades of evidence High quality: Further research is very unlikely to change our confidence in the estimate of effect. Moderate quality: Further research is likely to have an important impact on our confidence in the estimate of effect and may change the estimate. Low quality: Further research is very likely to have an important impact on our confidence in the estimate of effect and is likely to change the estimate. Very low quality: We are very uncertain about the estimate.				

* The basis for the assumed risk (e.g. the median control group risk across studies) is provided in footnotes. The corresponding risk (and its 95% confidence interval) is based on the assumed risk in the comparison group and the relative effect of the intervention (and its 95% CI). CI: Confidence interval; E2: beta-estradiol; BPA: bisphenol-A; MCF-7:breast cancer cells. ^1^ despite unclear risk of bias for certain domains in both studies, it was decided not to downgrade the quality of evidence for this reason. ^2^ downgraded 1 level for serious heterogeneity. ^3^ upgraded 2 levels for very large effect size. ^4^ despite unclear risk of bias for certain domains, it was decided not to downgrade the quality of evidence for this reason. ^5^ downgraded 1 level for imprecision, as only one study was included. ^6^ upgraded 1 level for large effect size.

## Supplementary Materials

The following are available online at https://www.mdpi.com/1996-1944/13/8/1843/s1, Table S1: Detailed assessment of included randomized trials with the RoB 2.0 tool (supplement to Table 2). Table S2: Summary of Findings Table and quality of the evidence regarding retainers’ effect on bisphenol-A (BPA) leaching.

## Appendix A

**Table A1:** Search strategy for study selection across databases.

No.	Electronic Database	Hits
1.	**Medline via PubMed**	15
	((essix) OR (vacuum formed aligner) OR (vacuum-formed aligner) OR (thermoplastic aligner) OR (aligner) OR (clear aligner) OR (invisalign) OR (vacuum-formed retainer) OR (vacuum formed retainer)) AND ((BPA) OR (BPA release) OR (bisphenol A) OR (bisphenol-A) OR (bisphenol-A release) OR (bisphenol A release) OR (monomer) OR (monomer release) OR (cytotoxicity) OR (estrogenicity))	
2.	**Scopus**	4
	((essix) OR (vacuum formed aligner) OR (vacuum-formed aligner) OR (thermoplastic aligner) OR (aligner) OR (clear aligner) OR (invisalign) OR (vacuum-formed retainer) OR (vacuum formed retainer)) AND ((BPA) OR (BPA release) OR (bisphenol A) OR (bisphenol-A) OR (bisphenol-A release) OR (bisphenol A release) OR (monomer) OR (monomer release) OR (cytotoxicity) OR (estrogenicity))	
3.	**Cochrane Central Register of Controlled Trials (CENTRAL)**	15
	((essix) OR (vacuum formed aligner) OR (vacuum-formed aligner) OR (thermoplastic aligner) OR (aligner) OR (clear aligner) OR (invisalign) OR (vacuum-formed retainer) OR (vacuum formed retainer)) AND ((BPA) OR (BPA release) OR (bisphenol A) OR (bisphenol-A) OR (bisphenol-A release) OR (bisphenol A release) OR (monomer) OR (monomer release) OR (cytotoxicity) OR (estrogenicity))	
4.	**Cochrane Database of Systematic Reviews (CDSR)**	0
	((essix) OR (vacuum formed aligner) OR (vacuum-formed aligner) OR (thermoplastic aligner) OR (aligner) OR (clear aligner) OR (invisalign) OR (vacuum-formed retainer) OR (vacuum formed retainer)) AND ((BPA) OR (BPA release) OR (bisphenol A) OR (bisphenol-A) OR (bisphenol-A release) OR (bisphenol A release) OR (monomer) OR (monomer release) OR (cytotoxicity) OR (estrogenicity))	
5.	**Google Scholar**	23
	((essix) OR (vacuum formed aligner) OR (vacuum-formed aligner) OR (thermoplastic aligner) OR (aligner) OR (clear aligner) OR (invisalign) OR (vacuum-formed retainer) OR (vacuum formed retainer)) AND ((BPA) OR (BPA release) OR (bisphenol A) OR (bisphenol-A) OR (bisphenol-A release) OR (bisphenol A release) OR (monomer) OR (monomer release) OR (cytotoxicity) OR (estrogenicity))	
6.	**Open Grey**	−
	(orthodontic aligner) AND (bisphenol)	0
	(orthodontic retainer) AND (bisphenol)	0
7.	**ClinicalTrials.gov (www.clinicaltrials.gov)**	−
	(orthodontic aligner) AND (bisphenol)	0
	(orthodontic retainer) AND (bisphenol)	0
8.	**National Research Register (ISRCTN: www.controlled-trials.com)**	−
	(orthodontic aligner) AND (bisphenol)	0
	(orthodontic retainer) AND (bisphenol)	0
