# Supplementary material for: Safety Considerations for Thermoplastic-Type Appliances Used as Orthodontic Aligners or Retainers. A Systematic Review and Meta-Analysis of Clinical and In-Vitro Research
Source: Materials (Basel). 2020 Apr 14;13(8):1843. doi: 10.3390/ma13081843 (PMC7215465; doi:10.3390/ma13081843)
Supplement: Supplementary file 1 [file materials-13-01843-s001.pdf]

materials

Supplementary Materials

Safety Considerations for Thermoplastic-Type Appliances Used as Orthodontic Aligners or Retainers. A Systematic Review and Meta-Analysis of Clinical and In-Vitro Research

Anna Iliadi [1,†], Despina Koletsi [2,†], Spyridon N. Papageorgiou [2] and Theodore Eliades [2,*]

[1] Department of Biomaterials, School of Dentistry, National and Kapodistrian University of Athens, 11527 Athens, Greece; annaeliades@gmail.com
[2] Clinic of Orthodontics and Pediatric Dentistry, Center of Dental Medicine, University of Zurich, 8032 Zurich, Switzerland; d.koletsi@gmail.com (D.K.); snpapage@gmail.com (S.N.P.)
* Correspondence: Theodore.Eliades@zzm.uzh.ch
† Equal author contribution

Table S1. Detailed assessment of included randomized trials with the RoB 2.0 tool (supplement to Table 2).

Domain	Reference	Raghavan 2017
1. Randomization process	1.1	PN
	1.2	PN
	1.3	PY
	Assessor's Judgement	High
2. Deviations from intended interventions	2.1	PY
	2.2	PY
	2.3	PY
	2.4	PY
	2.5	NI
	2.6	NI
	2.7	NI
	Assessor's Judgement	High
3. Mising outcome data	3.1	PY
	3.2	NA
	3.3	NA
	3.4	NA
	Assessor's judgement	Low
4. Measurement of the outcome	4.1	PN
	4.2	PN
	4.3	NI
	4.4	PY
	4.5	PN
	Assessor's Judgement	Some concerns
5. Selection of the reported result	5.1	NI
	5.2	PN
	5.3	NI
	Assessor's Judgement	Some concerns
Overall	Assessor's Judgement	High
–	General Note	No pre- registered protocol of the trial is publicly available

Y, yes; PY, probably yes; N, no; PN, probably no; NI, no information; NA, not applicable.

Table S2. Summary of Findings Table and quality of the evidence regarding retainers' effect on bisphenol-A (BPA) leaching.

BPA Levels (Whole Saliva Stimulation)

Patient or population: Patients in retention phase (post- orthodontic treatment)
Design: Randomized Controlled Trial
Interventions: VFRs or Hawley heat-cured or Hawley chemically-cured

**Outcomes**	**Illustrative comparative risks* (95% CI)**	**No of Participants (studies)**		**Quality of the evidence (GRADE)**
	Corresponding risk	–	–	–
–	VFR/Hawley heat cured/Hawley chemically cured	–	–	–
BPA levels (VFR vs Hawley heat cured); 7 days	The mean BPA levels (VFR vs Hawley heat cured); 7 days; in the intervention groups was 2.38 higher (1.47 to 3.29 higher)	30 (1 study)	⊕⊕⊖⊖ low[1]	–
BPA levels (VFR vs Hawley chemically cured); 7 days	The mean BPA levels (VFR vs Hawley chemically cured); 7 days; in the intervention groups was 2.38 higher (1.47 to 3.29 higher)	30 (1 study)	⊕⊕⊖⊖ low[1]	–
BPA levels (Hawley heat cured vs Hawley chemically cured); 7 days	The mean BPA levels (Hawley heat cured vs Hawley chemically cured); 7 days; in the intervention groups was 0.0035 lower (0.0037 to 0.0032 lower)	30 (1 study)	⊕⊕⊖⊖ low[1]	–
BPA levels (VFR vs Hawley heat cured); 30 days	The mean BPA levels (VFR vs Hawley heat cured); 30 days in the intervention groups was 0.20 higher (0.16 to 0.25 higher)	30 (1 study)	⊕⊕⊖⊖ low[1]	–
BPA levels (VFR vs Hawley chemically cured); 30 days	The mean BPA levels (VFR vs Hawley chemically cured); 30 days in the intervention groups was 0.20 higher (0.15 to 0.24 higher)	30 (1 study)	⊕⊕⊖⊖ low[1]	–
BPA levels (Hawley heat cured vs Hawley chemically cured); 30 days	The mean BPA levels (Hawley heat cured vs Hawley chemically cured); 30 days in the intervention groups was 0.009 lower (0.010 to 0.007 lower)	30 (1 study)	⊕⊕⊖⊖ low[1]	–

*The basis for the assumed risk (e.g. the median control group risk across studies) is provided in footnotes. The corresponding risk (and its 95% confidence interval) is based on the assumed risk in the comparison group and the relative effect of the intervention (and its 95% CI). CI: Confidence interval; BPA, bisphenol-A; VFR, vacuum-formed retainer

GRADE Working Group grades of evidence
High quality: Further research is very unlikely to change our confidence in the estimate of effect.
Moderate quality: Further research is likely to have an important impact on our confidence in the estimate of effect and may change the estimate.

Low quality: Further research is very likely to have an important impact on our confidence in the estimate of effect and is likely to change the estimate.<br>Very low quality: We are very uncertain about the estimate.
[1] downgraded 2 levels due to serious risk of bias in certain critical domains

© 2019 by the authors. Submitted for possible open access publication under the terms and conditions of the Creative Commons Attribution (CC BY) license (http://creativecommons.org/licenses/by/4.0/).
